# Local Unitary Equivalence of Quantum States Based on the Tensor Decompositions of Unitary Matrices

**DOI:** 10.3390/e25081139

**Published:** 2023-07-29

**Authors:** Jing Wang, Xiaoqi Liu, Li Xu, Ming Li, Lei Li, Shuqian Shen

**Affiliations:** College of Science, China University of Petroleum, Qingdao 266580, China; wangjing3310@163.com (J.W.); qii1217@163.com (X.L.); b22090008@s.upc.edu.cn (L.X.); lileiupc@126.com (L.L.); shuqianshen@126.com (S.S.)

**Keywords:** local unitary equivalence, quantum states, quantum entanglement

## Abstract

Since two quantum states that are local unitary (LU) equivalent have the same amount of entanglement, it is meaningful to find a practical method to determine the LU equivalence of given quantum states. In this paper, we present a valid process to find the unitary tensor product decomposition for an arbitrary unitary matrix. Then, by using this process, the conditions for determining the local unitary equivalence of quantum states are obtained. A numerical verification is carried out, which shows the practicability of our protocol. We also present a property of LU invariants by using the universality of quantum gates which can be used to construct the complete set of LU invariants.

## 1. Introduction

Quantum entanglement is one of the most extraordinary features in quantum information science, and quantum entangled states have become the most important physical resource [1]. In particular, multipartite quantum entanglement plays key roles in the rapidly developing field of quantum information science, for example, in one-way quantum computing, quantum error correction, and quantum secret sharing [2,3]. However, it is more difficult to understand multipartite mixed states with nonlocal properties. Fortunately, the entanglement (or the local hidden variable models) of quantum states remains unchanged under local unitary (LU) transformations. In addition, local operations and classical communication (LOCC) equivalence states are interconvertible also by local unitary transformations [4]. Therefore, it is very important to determine whether or not two states are LU equivalent.

**Definition 1.**  *Let ρ and $\tilde{\rho }$ be two states in general $H_{1}⊗H_{2}⊗\cdots H_{N}$ quantum systems with dim$H_{i}=d_{i}$, i=1,2,⋯,N. They are LU equivalent if*

$$\tilde{\rho }=\left(U_{1}⊗U_{2}⊗\cdots U_{N}\right)\rho {\left(U_{1}⊗U_{2}⊗\cdots U_{N}\right)}^{†}$$

*for some unitary operators $U_{i}$, i=1,2,⋯,N, where † denotes transpose and conjugate.*

At present, there are many results on LU equivalence and LU invariants [4,5,6,7,8,9,10,11,12,13,14,15,16,17,18,19,20,21,22,23,24]. In this paper, we present a practical method to find the unitary tensor decomposition of an arbitrary unitary matrix. Then, we derive a local unitary equivalence strategy for arbitrary quantum states with non-degenerate density matrices from the point of view of block matrix and unitary matrix tensor decomposition. Exact examples are analyzed numerically. We also present a property of LU invariants which can lead to the construction of a complete set of LU invariants.

## 2. Unitary Tensor Decomposition of an Arbitrary Unitary Matrix

In this section, we will present a sufficient and necessary condition for the existence of the unitary tensor decomposition of an arbitrary unitary matrix. We start with the bipartite decomposition.

### 2.1. The Decomposition Scheme of W=U⊗V

Set W=U⊗V, with *U* and *V* as 2×2 and d×d matrices, respectively. Set

$$U=\left[\begin{array}{cc} u_{11} & u_{12}\\ u_{21} & u_{22} \end{array}\right]$$

with $u_{kl}$ as the entries, and

(1) $$W=\left[\begin{array}{cc} W_{11} & W_{12}\\ W_{21} & W_{22} \end{array}\right]$$

as the block representation of *W*. Then, according to the tensor product of matrices, one obtains

$$W=U⊗V=\left[\begin{array}{cc} u_{11}V & u_{12}V\\ u_{21}V & u_{22}V \end{array}\right]$$

We have $W_{kl}W_{mn}^{†}=u_{kl}VV^{†}u_{mn}^{†}$, where k,l,m,n∈{1,2}. Furthermore, we can obtain $tr(W_{kl}W_{kl}^{†})$$=d|u_{kl}{|}^{2}$, when k=m, l=n. Thus,

(2) $$u_{kl}=\sqrt{\frac{tr(W_{kl}W_{kl}^{†})}{d}}\cdot e^{i\theta_{kl}},$$

where $e^{i\theta_{kl}}$ is the complex phase of $u_{kl}$, k,l={1,2}.

Thus, we set

(3) $$W=\left[\begin{array}{cc} {\tilde{u}}_{11} & {\tilde{u}}_{12}\\ {\tilde{u}}_{21} & {\tilde{u}}_{22} \end{array}\right]⊗e^{i\theta_{11}}V\equiv \tilde{U}⊗\tilde{V},$$

with $\tilde{U}=e^{-i\theta_{11}}U$ and $\tilde{V}=e^{i\theta_{11}}V$. Without loss of generality, we set $tr(W_{11}W_{11}^{†})\neq 0$. Combining (1) and (3), we can obtain

(4) $$\tilde{V}=e^{i\theta_{11}}\cdot V=\frac{W_{11}}{{\tilde{u}}_{11}},$$

where ${\tilde{u}}_{11}=\sqrt{\frac{tr(W_{11}W_{11}^{†})}{d}}$. We can further derive that for the remaining entries of ${\tilde{u}}_{kl}$ with kl={12,21,22},

(5) $${\tilde{u}}_{kl}=\frac{{\left(W_{kl}\right)}_{11}}{{\tilde{v}}_{11}},$$

where ${\tilde{v}}_{11}$ is the (1,1) entry of $\tilde{V}$, which is assumed to be nonzero. We also obtain that matrices $W_{kl}$ must be the scalar multiplications of $\tilde{V}$ for any k,l∈{1,2}.

For unitary matrix W=U⊗V, where *U* and *V* are arbitrary $d_{1}\times d_{1}$ and $d_{2}\times d_{2}$ unitary matrices, we can also find unitary matrices $\tilde{U}$ and $\tilde{V}$ such that $W=\tilde{U}⊗\tilde{V}$ by using the same method.

To summarize, let *W* be any $d_{1}d_{2}\times d_{1}d_{2}$ unitary matrix with block representation $W=(W_{kl})$, where $k,l\in \{1,2,\cdots ,d_{1}\}$ and $W_{kl}$ are $d_{2}\times d_{2}$ matrices. According to the above analysis, we directly derive the following theorem.

**Theorem 1.**  *If $W_{kl}$ is a scalar multiplication of a unitary matrix for any $k,l\in \{1,2,\cdots ,d_{1}\}$, then we can always derive the tensor product decomposition $W=\tilde{U}⊗\tilde{V}$, where $\tilde{U}$ and $\tilde{V}$ are $d_{1}\times d_{1}$ and $d_{2}\times d_{2}$ unitary matrices, respectively. We can always select one of $tr(W_{kl}W_{kl}^{†})\neq 0$. Without loss of generality, we set $tr(W_{11}W_{11}^{†})\neq 0$. The entries of $\tilde{U}$ and $\tilde{V}$ are given by*

(6) $$\begin{array}{ccc} {\tilde{u}}_{11} & = & \sqrt{\frac{tr(W_{11}W_{11}^{†})}{d_{1}}};\\ \tilde{V} & = & \frac{W_{11}}{{\tilde{u}}_{11}};\\ {\tilde{u}}_{kl} & = & \frac{{\left(W_{kl}\right)}_{11}}{{\tilde{v}}_{11}},k,l\in \left\{1,2,\cdots d_{1}\right\},kl\neq 11. \end{array}$$

### 2.2. The Decomposition Scheme of W=Q⊗U⊗V

To simplify the expression, we set Q,U and *V* as 2×2 matrices with entries $q_{kl},u_{kl}$ and $v_{kl}$, respectively. We then consider W=Q⊗U⊗V=Q⊗P, and set P=U⊗V. We obtain

$$W=\left[\begin{array}{cc} q_{11}P & q_{12}P\\ q_{21}P & q_{22}P \end{array}\right]=\left[\begin{array}{cc} W_{11} & W_{12}\\ W_{21} & W_{22} \end{array}\right],$$

where *W_kl_* are the block matrices of *W*, and

$$P=\left[\begin{array}{cc} u_{11}V & u_{12}V\\ u_{21}V & u_{22}V \end{array}\right]=\left[\begin{array}{cccc} p_{11} & p_{12} & p_{13} & p_{14}\\ p_{21} & p_{22} & p_{23} & p_{24}\\ p_{31} & p_{32} & p_{33} & p_{34}\\ p_{41} & p_{42} & p_{43} & p_{44} \end{array}\right],V=\left[\begin{array}{cc} v_{11} & v_{12}\\ v_{21} & v_{22} \end{array}\right].$$

We can obtain $W_{kl}W_{mn}^{†}=q_{kl}PP^{†}q_{mn}^{†}$, and $tr\left(W_{kl}W_{kl}^{†}\right)=2\left|q_{kl}\right|$, for k,l,m,n∈{1,2}. Thus,

$$q_{kl}=\sqrt{\frac{tr(W_{kl}W_{kl}^{†})}{4}}\cdot e^{i\theta_{kl}},$$

where $e^{i\theta_{kl}}$ is the complex phase of $q_{kl}$, k,l∈{1,2}.

Without loss of generality, one still sets $tr(W_{11}W_{11}^{†})\neq 0$. We then set

(7) $$\begin{array}{ccc} {\tilde{q}}_{11} & = & \sqrt{\frac{tr(W_{11}W_{11}^{†})}{4}};\\ \tilde{P} & = & \frac{W_{11}}{{\tilde{q}}_{11}};\\ {\tilde{q}}_{kl} & = & \frac{{\left(W_{kl}\right)}_{11}}{{\tilde{p}}_{11}},kl=\left\{12,21,22\right\}. \end{array}$$

Thus, we have

$$W=e^{i\theta_{11}}\tilde{Q}⊗P=\tilde{Q}⊗e^{i\theta_{11}}P=\tilde{Q}⊗\tilde{P}.$$

Then, by using the decomposition scheme in Section 2.1 for $\tilde{P}$, one finally obtains

$$W=\tilde{Q}⊗\tilde{U}⊗\tilde{V}.$$

One can then derive the decomposition scheme of $W=U_{1}⊗U_{2}⊗\cdots ⊗U_{N}$ in the same way.

### 2.3. Numerical Verification

We use the rand() function in MATLAB to generate a random matrix [25,26]. This function can generate random numbers between 0 and 1 according to a uniform distribution. Then, using the singular value decomposition of this matrix, a random unitary matrix can be obtained.

**Example 1.**  *As an example, let us consider the tensor decomposition of the following unitary matrix:*

$$W=\left[\begin{array}{cccc} 0.439154-0.260657i & 0.489398+0.00346013i & 0.43702-0.263586i & 0.489097+0i\\ 0.42086-0.249799i & -0.510671-0.00361053i & 0.418815-0.252606i & -0.510356+0i\\ 0.438872-0.26049i & 0.489085+0.00345791i & -0.4373+0.263755i & -0.489411+0i\\ 0.42059-0.249639i & -0.510344-0.00360822i & -0.419084+0.252768i & 0.510684+0i \end{array}\right],$$

*which is generated randomly by $U_{1}$ and $U_{2}$, i.e., $W=U_{1}⊗U_{2}$, where*

$$U_{1}=\left[\begin{array}{cc} 0.7073157717804006+0.005000843737265154i & 0.70688003971863\\ 0.7068623728603339+0.004997638129189724i & -0.7073334499706543 \end{array}\right],$$

*and*

$$U_{2}=\left[\begin{array}{cc} 0.6182373567761775-0.3728863629370793i & 0.69190919275723i\\ 0.5924837578103638-0.3573532254686829i & -0.721984535137726 \end{array}\right].$$

*According to the previous analysis, W can be decomposed as W=U⊗V with*

$$U=\left[\begin{array}{cc} 0.7073+0.0000i & 0.7069-0.0050i\\ 0.7069-0.0000i & -0.7073+0.0050i \end{array}\right],$$

*and*

$$V=\left[\begin{array}{cc} 0.6209-0.3685i & 0.6919+0.0049i\\ 0.5950-0.3532i & -0.7220-0.0051i \end{array}\right].$$

**Example 2.**  *We consider a 8×8 unitary matrix $W=Y_{1}+Y_{2}i$, with*

$$Y_{1}=\left[\begin{array}{cccccccc} 0.4652 & 0.1795 & 0.4922 & 0.1948 & 0.4231 & 0.1783 & 0.4355 & 0.1881\\ 0.1932 & -0.430 & 0.2045 & -0.4691 & 0.1758 & -0.4292 & 0.1809 & -0.4529\\ 0.4890 & 0.1886 & -0.4683 & -0.1854 & 0.4447 & 0.1874 & -0.4144 & -0.1790\\ 0.2031 & -0.4541 & -0.1945 & 0.4463 & 0.1847 & -0.4511 & -0.1721 & 0.4309\\ 0.4278 & 0.1650 & 0.4526 & 0.1792 & -0.4601 & -0.1939 & -0.4736 & -0.2046\\ 0.1777 & -0.3973 & 0.1880 & -0.4313 & -0.1911 & 0.4668 & -0.1967 & 0.4925\\ 0.4496 & 0.1735 & -0.4307 & -0.1705 & -0.4836 & -0.2038 & 0.4506 & 0.1946\\ 0.1868 & -0.4176 & -0.1789 & 0.4104 & -0.2009 & 0.4906 & 0.1872 & -0.4686 \end{array}\right],$$

*and*

$$Y_{2}=\left[\begin{array}{cccccccc} -0.0560 & -0.0753 & -0.0158 & -0.0624 & 0.0814 & -0.0157 & 0.1241 & 0\\ -0.0233 & 0.1813 & -0.0066 & 0.1501 & 0.0338 & 0.0377 & 0.0516 & 0\\ -0.0588 & -0.0792 & 0.0151 & 0.0593 & 0.0855 & -0.0165 & -0.1181 & 0\\ -0.0244 & 0.1906 & 0.0063 & -0.1428 & 0.0355 & 0.0396 & -0.0490 & 0\\ -0.0515 & -0.0693 & -0.0146 & -0.0573 & -0.0885 & 0.0170 & -0.1350 & 0\\ -0.0214 & 0.1667 & -0.0060 & 0.1380 & -0.0368 & -0.0410 & -0.0561 & 0\\ -0.0541 & -0.0728 & 0.0138 & 0.0546 & -0.0930 & 0.0179 & 0.1284 & 0\\ -0.0225 & 0.1753 & 0.0058 & -0.1313 & -0.0386 & -0.0431 & 0.0533 & 0 \end{array}\right],$$

*which is randomly generated by*

$$U_{1}=\left[\begin{array}{cc} 0.701056-0.224367i & 0.67689\\ 0.644678-0.206324i & -0.736084 \end{array}\right],$$

$$U_{2}=\left[\begin{array}{cc} 0.686641-0.06032i & 0.72449\\ 0.721711-0.0634009i & -0.689285 \end{array}\right],$$

$$U_{3}=\left[\begin{array}{cc} 0.888143+0.253079i & 0.383606\\ 0.36892+0.105125i & -0.923497 \end{array}\right].$$

*After numerical verification, the unitary tensor decomposition W=Q⊗U⊗V can be obtained with*

$$Q=\left[\begin{array}{cc} 0.7361-0.0000i & 0.6447+0.2063i\\ 0.6769-0.0000i & -0.7011-0.2244i \end{array}\right].$$

$$U=\left[\begin{array}{cc} 0.6893+0.0000i & 0.7217+0.0634i\\ 0.7245-0.0000i & -0.6866-0.0603i \end{array}\right],$$

$$V=\left[\begin{array}{cc} 0.9169-0.1103i & 0.3537-0.1485i\\ 0.3809-0.0458i & -0.8515+0.3574i \end{array}\right].$$

*The Matlab code is supplied in the Supplementary Materials.*

## 3. Determine the LU Equivalence of Non-Degenerate Quantum States

The key to investigating the local unitary equivalence of quantum states lies in the unitary tensor decomposition of the corresponding unitary matrix. In this section, we present a general method to determine the LU equivalence of any pair of non-degenerate quantum states by the unitary tensor decomposition protocol derived in the above section.

Let ρ and $\tilde{\rho }$ be the density matrices of two states in quantum systems $H_{1}⊗H_{2}⊗\cdots H_{N}$ with dim$H_{i}=d$, i=1,2,⋯,N. We assume that both ρ and $\tilde{\rho }$ are non-degenerate. We further set that ρ and $\tilde{\rho }$ have the same eigenvalues, which is the necessary condition for the LU equivalence of the two density matrices. Let $\rho =X\Sigma X^{†}$ and $\tilde{\rho }=Y\Sigma Y^{†}$ be the spectral decomposition of ρ and $\tilde{\rho }$. Thus, there is a unitary matrix $W=YX^{†}$ such that

$$\tilde{\rho }=W\rho W^{†}.$$

To certify that ρ and $\tilde{\rho }$ are local unitary equivalent, one needs to further find unitary matrices $U_{i}$, i=1,2,⋯,N such that

$$W=U_{1}⊗U_{2}⊗\cdots U_{N}.$$

In the following, we consider bipartite quantum systems as an example. The processes of judging the local unitary equivalence of quantum states are as follows:

[1] Check whether the density matrices ρ and σ of quantum states are non-degenerate and whether they have the same eigenvalues;

[2] Find the spectral decompositions $\rho =X\Sigma X^{†}$ and $\tilde{\rho }=Y\Sigma Y^{†}$. Compute $W=YX^{†}$;

[3] Determine whether *W* can be decomposed into the tensor product of two unitary matrices, such as W=U⊗V.

**Example 3.**  *We consider two quantum states*

$$\rho =\left[\begin{array}{cccc} 0.2 & 0 & 0 & 0\\ 0 & 0.2743 & 0.0429 & -0.0086\\ 0 & 0.0429 & 0.2286 & 0.0143\\ 0 & -0.0086 & 0.0143 & 0.2971 \end{array}\right],$$

$$\sigma =\left[\begin{array}{cccc} 0.2648 & -0.0360 & -0.0228 & -0.0217\\ -0.0360 & 0.2225 & 0.0212 & -0.0011\\ -0.0228 & 0.0212 & 0.2369 & -0.0369\\ -0.0217 & -0.0011 & -0.0369 & 0.2759 \end{array}\right]$$

*with spectral decompositions $\rho =X\Sigma X^{†}$ and $\sigma =Y\Sigma Y^{†}$, respectively, where*

$$\Sigma =\left[\begin{array}{cccc} 0.2 & 0 & 0 & 0\\ 0 & 0.2 & 0 & 0\\ 0 & 0 & 0.3 & 0\\ 0 & 0 & 0 & 0.3 \end{array}\right],$$

$$X=\left[\begin{array}{cccc} 1 & 0 & 0 & 0\\ 0 & -0.507092552837110 & 0.609449400220044 & 0.609449400220044\\ 0 & 0.845154254728517 & 0.212930374147564 & 0.490280472260179\\ 0 & -0.169030850945703 & -0.763696329922311 & 0.623054160640762 \end{array}\right],$$

$$Y=\left[\begin{array}{cccc} -0.585389075528045 & -0.098738906989822 & -0.087850024051642 & -0.799907889555417\\ -0.490973763578605 & -0.730809679765713 & -0.109038680416598 & 0.461489481582411\\ -0.494334763695753 & 0.621997602780637 & -0.502988683425162 & 0.340227141602823\\ -0.414605276295110 & 0.263223901540746 & 0.852874740975106 & 0.177257774815572 \end{array}\right].$$

*Then, the matrix $W=YX^{†}$ can be decomposed into the tensor product of two unitary matrices, i.e., W=U⊗V, where*

$$U=\left[\begin{array}{c} 0.7640,0.6452\\ 0.6452,-0.7640 \end{array}\right],$$

$$V=\left[\begin{array}{c} -0.7662,-0.6426\\ -0.6426,0.7662 \end{array}\right].$$

*Therefore, the quantum states ρ and σ are local unitary equivalent.*

## 4. LU Invariants

Let f(ρ) be a function of density matrix $\rho \in H_{A}⊗H_{B}$. In this section, we set $dimH_{A}=dimH_{B}=d=2^{n}$. If *f* is an LU invariant, then for any quantum state ρ and unitary matrices *U* and *V*, it satisfies

$$f\left(\rho^{\prime }\right)=f\left(\rho \right),$$

where $\rho^{\prime }=U⊗V\rho U^{†}⊗V^{†}$. Such kinds of functions contain polynomial local unitary invariants, rational local unitary invariants and so on [24]. Let *G* be the set of all “single-qubit” and CNOT gates. According to the universality of quantum gates, an arbitrary unitary operation on *n* qubits can be implemented using a circuit containing $O(n^{2}4^{n})$ unitary operators in *G*. Then, for any unitary matrices *U* and *V*, there exist unitary matrices $U_{i}\in G$ and $V_{i}\in G$, i=1,2,…,k, such that $U=U_{1}⊗U_{2}⊗\cdots ⊗U_{k}$ and $V=V_{1}⊗V_{2}⊗\cdots ⊗V_{k}$, respectively.

**Theorem 2.**  *A function f is an LU invariant if and only if f is invariable under LU operations of the form $U_{i}⊗I$ and $I⊗V_{i}$ for all $U_{i}\in G$ and $V_{i}\in G$.*

**Proof.**  The “only if” is obvious. For the if part, one has to prove that the following equation

$$f\left(\rho^{\prime }\right)=f\left(U⊗V\rho U^{†}⊗V^{†}\right)=f\left(\rho \right)$$

holds. Suppose *U* and *V* can be rewritten as $U=U_{1}⊗U_{2}⊗\cdots ⊗U_{k_{1}}$ and $V=V_{1}⊗V_{2}⊗\cdots ⊗V_{k_{2}}$. One can always set $k_{1}=k_{2}=k$. Otherwise, the identity matrix *I* can be used as a complement. We have

$$\begin{array}{cc} f(\rho^{\prime }) & =f(U⊗V\rho U^{†}⊗V^{†})\\ \; & =f(\left(U_{1}⊗V_{1}\right)\left(U_{2}⊗V_{2}\right)\cdots \left(U_{k}⊗V_{k}\right)\rho {\left(U_{k}⊗V_{k}\right)}^{†}\cdots {\left(U_{2}⊗V_{2}\right)}^{†}{\left(U_{1}⊗V_{1}\right)}^{†})\\ \; & =f(\left(U_{1}⊗I\right)\left(I⊗V_{1}\right)\left(U_{2}⊗V_{2}\right)\cdots \left(U_{k}⊗V_{k}\right)\rho {\left(U_{k}⊗V_{k}\right)}^{†}\cdots {\left(U_{2}⊗V_{2}\right)}^{†}{\left(I⊗V_{1}\right)}^{†}{\left(U_{1}⊗I\right)}^{†})\\ \; & =f(\left(I⊗V_{1}\right)\left(U_{2}⊗V_{2}\right)\cdots \left(U_{k}⊗V_{k}\right)\rho {\left(U_{k}⊗V_{k}\right)}^{†}\cdots {\left(U_{2}⊗V_{2}\right)}^{†}{\left(I⊗V_{1}\right)}^{†})\\ \; & =\cdots \\ \; & =f(\rho ), \end{array}$$

which ends the proof of the theorem. □

## 5. Conclusions

In this paper, we have studied the local unitary equivalence of quantum systems from the perspective of unitary matrix tensor decomposition. We have presented a detailed process to find the unitary matrices in the tensor decomposition of an arbitrary tensor-factorable unitary matrix. We have also derived a property of LU invariants that may be used to find a complete set of LU invariants.

It should be noted that our schemes are convenient to discuss the local unitary equivalence when the number and the dimension of the subsystems are small. As quantum systems get more complex, the amount of computations increases exponentially. Therefore, we need to further find more convenient and efficient strategies to judge the local unitary equivalence of multipartite high-dimensional quantum systems.

## Data Availability

All data generated or analysed during this study are included in this published article.

## Supplementary Materials

The following supporting information can be downloaded at: https://www.mdpi.com/article/10.3390/e25081139/s1.
